# Switching from high-fat diet to normal diet ameliorate BTB integrity and improve fertility potential in obese male mice

**DOI:** 10.1038/s41598-023-41291-2

**Published:** 2023-08-29

**Authors:** Wenjing Zhang, Zhenhua Tian, Xiangyu Qi, Pengcheng Chen, Qian Yang, Qingbo Guan, Jifeng Ye, Chunxiao Yu

**Affiliations:** 1 Department of Endocrinology, Shandong Provincial Hospital Affiliated to Shandong First Medical University, Shandong Provincial Hospital, Shandong University, Jinan, 250021 Shandong China; 2 Key Laboratory of Endocrine Glucose and Lipids Metabolism and Brain Aging (Shandong First Medical University), Ministry of Education, Shandong, China; 3 Shandong Key Laboratory of Endocrinology and Lipid Metabolism, Jinan, 250021 Shandong China; 4 Shandong Institute of Endocrine and Metabolic Diseases, Jinan, 250021 Shandong China; 5 Shandong Engineering Laboratory of Prevention and Control for Endocrine and Metabolic Diseases, Jinan, 250021 Shandong China; 6 Department of Endocrinology and Metabolism, The Second People’s Hospital of Liaocheng, Shandong, 252601 China

**Keywords:** Endocrinology, Medical research, Pathogenesis

## Abstract

Obesity is a prominent risk factor for male infertility, and a high-fat diet is an important cause of obesity. Therefore, diet control can reduce body weight and regulate blood glucose and lipids, but it remains unclear whether it can improve male fertility and its mechanism. This study explores the effects of switching from a high-fat diet (HFD) to a normal diet (ND) on the fertility potential of obese male mice and its related mechanisms. In our study, male mice were separated into three groups: normal diet group (NN), continuous high-fat diet group (HH), and return to normal diet group (HN). The reproductive potential of mice was tested through cohabitation. Enzymatic methods and ELISA assays were used to measure metabolic indicators, follicle-stimulating hormone (FSH) levels and intratesticular testosterone levels. Transmission electron microscopy and immunofluorescence with biotin tracers assessed the integrity of the blood-testis barrier (BTB). Malondialdehyde (MDA), superoxide dismutase (SOD), and reactive oxygen species (ROS) were inspected for the assessment of oxidative stress. The expression and localization of BTB-related proteins were detected through the immunoblot and immunofluorescence. The mice in the high-fat diet group indicated increased body weight and epididymal fat weight, elevated serum TC, HDL, LDL, and glucose, decreased serum FSH, and dramatic lipid deposition in the testicular interstitium. Analysis of fertility potential revealed that the fertility rate of female mice and the number of pups per litter in the HH group were significantly reduced. After the fat intake was controlled by switching to a normal diet, body weight and epididymal fat weight were significantly reduced, serum glucose and lipid levels were lowered, serum FSH level was elevated and the deposition of interstitial lipids in the testicles was also decreased. Most significantly, the number of offspring of male mice returning to a normal diet was significantly increased. Following further mechanistic analysis, the mice in the sustained high-fat diet group had disrupted testicular BTB integrity, elevated levels of oxidative stress, and abnormal expression of BTB-related proteins, whereas the restoration of the normal diet significantly ameliorated the above indicators in the mice. Our study confirms diet control by switching from a high-fat diet to a normal diet can effectively reduce body weight, ameliorate testicular lipotoxicity and BTB integrity in male mice, and improve fertility potential, providing an effective treatment option for obese male infertility.

## Introduction

Obesity and overweight are caused by abnormal or excessive fat accumulation, possibly impairing health. They are metabolic disorders influenced by lifestyle factors like physical activity, types and amounts of food consumed, and genetic^1,2^. In recent years, the number of overweight people has rapidly grown, culminating in a global obesity pandemic^3^. A decline in male fertility has accompanied this rise in obesity rates^4^. Numerous studies have indicated that obesity and being overweight are influential factors in low fertility rates among males^5–8^, impairing the male sexual health and fertility by affecting erectile function and semen parameters, respectively^8^. The previous study has proven that animal models of diet-induced obesity had lowered testosterone levels^9^ and abnormal sperm parameters^10–12^, including reduced sperm viability, decreased sperm count and growing sperm malformations, leading to decreased fertility.

Dietary modification is considered the cornerstone of weight control. The international consensus advocates that the most effective way of losing weight is through reducing calories by controlling the source of fat. In contrast to high-calorie diet regimens, the intake of low-calorie diets leads to short-term weight loss in obese individuals, as well as improved glucose control and lipid metabolism^13,14^. Additionally, some evidence suggests that weight loss through exercise, lifestyle changes, or surgery can improve serum testosterone levels and sperm counts, aiding male fertility^15,16^. In several prospective studies and randomised controlled trials, weight loss through low-calorie or low-fat diets increased erectile function and testosterone levels in men^17–19^. However, the mechanisms of weight loss on low-calorie diets improving male fertility are incompletely characterized.

A specialised junction between adjacent Sertoli cells close to the basement membrane of the seminiferous epithelium forms the blood–testis barrier (BTB), deemed one of the tightest epithelial barriers in the entire body^20,21^. Damage to the BTB can cause germ cell loss, decreased sperm counts, and male infertility^22^. In addition, Sertoli cells (SCs) are the predominant part of the BTB. A tight junction, adherens junction, and gap junction are formed between the SCs in BTB, which comprise various associated proteins such as ZO-1, and Occludin^8,23^. The FSH affects SCs proliferation, maturity, and function and regulates structural genes critical to organising cell–cell junctions and metabolizing nutrition delivered by SCs to germ cells^24–29^. Yet it is unclear whether returning to a normal diet can have an effect on FSH and its regulated BTB structure.

A number of studies have demonstrated that oxidative stress is able to impair BTB integrity^29–31^. The body experiences oxidative stress when reactive oxygen species (ROS) are produced at a higher rate than antioxidant defenses. Moreover, obesity is a major contributor to systemic oxidative stress^32^. Substantial evidence suggests ROS-mediated injury to the spermatozoa is the primary contributing factor in 30–80% of infertility cases^33,34^. Our previous work has indicated that HFD mice cause damage to the BTB following increased systemic oxidative stress, thereby diminishing fertility in male mice^35^. However, it remains unclear whether switching from a high-fat to a normal diet can alleviate BTB damage by improving oxidative stress and thus benefitting male fertility. In this study, to elucidate the effect of diet control by switching from HFD to ND regarding the fertility of obese male mice, an obese mouse model was created by feeding it HFD, while a lowered-fat mouse model was established by withdrawing the HFD and feeding it ND. Additionally, the alterations in testicular ultra-microstructure, testicular oxidative stress levels, BTB integrity, and related proteins were further evaluated to investigate the effects and mechanisms of controlled fat intake on male fertility.

## Materials and methods

### Animals and diets

The study was carried out in compliance with the ARRIVE guidelines (Animal Research: Reporting of In Vivo Experiments). All animal experiments were approved by the Animal Ethics Committee of Shandong Provincial Hospital and conducted per the requirements of the Shandong Provincial Hospital Animal Protection and Utilization Committee. Animal Ethics Approval No: 2016-08. Vital River Corporation (Beijing, China) provided 7-week-old male and 9-week-old female C57BL/6 mice for this experiment, which were housed in rooms controlled in terms of temperature at 22–25 °C and 12-h light/dark cycle.

In a randomization procedure, male mice were randomly placed into two groups and given either of these two diets for eight weeks: the normal diet group was fed a standard diet (D12450B, Research Diet) in which 10% of the calories were from fat, while the high-fat diet group was given a high-fat diet (D12492, Research Diet) in which 60% of the calories came from fat. The energy availability of the two diets is shown in Table S1. For further mechanistic analysis, mice in the normal diet group continued to be fed with the standard diet for 8 weeks (NN, n = 10). Mice in the high-fat diet group were randomly divided into two subgroups and continued to be fed with different diets for 8 weeks: (a) maintain feeding with the high-fat diet (HH = 10) or (b) restore to the normal diet (HN = 10). The body weight of the mice was monitored weekly at 5 p.m. throughout the rearing period (acclimation weeks and the following 16 weeks). At the end of the experiment, mice were sacrificed at 16 weeks. The design of the animal models is illustrated in Figure S1.

After being fasted for 8 h, all mice were anesthetized by intraperitoneal injection of Avertin (280 mg/kg). After anesthesia, the whole blood of mice was collected from the eyeballs and centrifuged twice at 3500 rpm for 15 min. Then the serum supernatant was stored at – 80 °C until assayed. Testis and epididymal fat were immediately separated and weighed. One testis was fixed in Modified Davidson’s fluid for morphological analysis and immunofluorescence, fixed in 2.5% glutaraldehyde and 1% osmium tetroxide for ultrastructure analysis, and the other testis was rapidly stored in liquid nitrogen for oil red staining, mRNA and protein analysis, and oxidative stress-related testing. Eventually, all mice were euthanized by CO_2_ inhalation.

### Reproductive ability assay

Female mice were randomly grouped based on their body weight at the 15th week, and the male mice were mated with two females randomly for five consecutive days between 5 pm and 8 am for the comparison of the reproductive capabilities of male mice fed differently. Every morning, female mice's vaginal plugs were examined to see if sexual intercourse had taken place. They were moved to another cage to be observed until the pups were born. Fertility potential was assessed by the number of pregnant females and by determining the number and size of litter delivered.

### Lipid parameter and sex hormone analysis

Routine enzyme measurements were performed on serum glucose and lipid profiles. A lipid metabolism analysis was conducted by measuring low-density lipoprotein cholesterol (LDL-cholesterol), high-density lipoprotein cholesterol (HDL-cholesterol), triglyceride (TG), and total cholesterol (TC) levels in automatic biochemical analyzer (Beckmen, AU5831, USA). Besides, We evaluated serum levels of follicle-stimulating hormone (FSH) using a commercially available ELISA kit supplied by Abnova.

### Oil Red O staining

To observe the extent of lipid deposition in the testis, we performed Oil Red O staining with frozen testicular sections. Oil Red O staining was carried out using an oil red dye from Changsha Guge Biotechnology according to the manufacturer's directions. Briefly, frozen sections (10 μm) of testis were fixed in slides with 95% ethanol for 10 s. Next, the samples were rinsed in distilled water for 10 s, washed in tap water for 2 min, and finally incubated in oil red dye for 15 min in the dark. The slides were then washed again with distilled water, and counter corroded with hematoxylin for 30 s.

### Haematoxylin and Eosin (H&E) staining

A series of graded ethanol solutions was consumed to dehydrate and embed paraffin-embedded testicular tissue samples to verify the histopathological changes. A microtome (Leica, USA) was used to section paraffin to a thickness of 4 μm, and the sections were then stained with H&E according to standard protocols. Finally, images are captured using a Carl Zeiss microscope to observe pathological alterations in testicular tissue.

### Malondialdehyde (MDA), superoxide dismutase (SOD), and ROS measurements

The ELISA kits for SOD (S0088) and MDA (S0131S) were all obtained from the Beyotime Institute of Biotechnology, as well as a ROS assay kit (E004-1-1) provided by Nanjing Jiancheng Bioengineering Institut. As directed by the manufacturer, MDA content, SOD activity, and ROS levels were measured using the ELISA kits, and these measurements were normalized to total protein content.

### Testicular transmission electron microscopy (TEM) analysis

Testis were dehydrated in increments of ethanol and acetone solutions, embedded in EPON 812, and sectioned with an LKB ultramicrotome to determine how they changed at the ultrastructural level. In the following steps, the testis were stained with uranyl acetate and dyed with lead citrate before being observed with a JEM-1200EX transmission electron microscope (JEOL, Japan).

### BTB integrity assay

The integrity of BTB was in vitro assessed using a biotin tracer and analyzed with a fluorescence microscope. Briefly, mice were anesthetized and then the testis were exposed. The gaps below the testicular tunica albuginea were injected with 50 μl of Biotin (10 mg/ml) and the animals were euthanized after 30 min. The tissues of mice testis were prepared in 10 μm frozen sections. After being fixed and blocked according to standard methods, frozen testicular sections were incubated with Alexa Fluor^®^ 568-conjugated streptavidin and DAPI for 2 h at room temperature. Finally, the biotin was visualized by fluorescence microscopy.

### Real-time quantitative PCR (qPCR) analyses

Based on the instructions of the manufacturer, RNA was isolated from 20mg of frozen testicular tissue using an RNA prep kit (LS1040, Super Total RNA Extraction Kit, Promega) and reverse-transcribed into cDNA (TaKaRaBio Inc., Japan). Genes for ZO-1, Occludin, N-cadherin, beta-Catenin, and Nectin2 were amplified using SYBR premix Ex Taq II (Takara Bio) in a TP850 Thermal Cycler Dice Real Time System Single (Takara, Japan). The primer sequences are listed in Table 1. Thermal cycling conditions were as follows: 5 min at 95 °C; 40 cycles of 10 s at 95 °C, 10 s at 60 °C, 10 s at 72 °C; melting curve from 95 to 60 °C; and 37 °C for 10 s.

**Table 1:** Primer sequences were used for the analysis of mRNA expression levels.

Gene	Forward primer	Size (bp)	Reverse primer	Size (bp)
ZO-1	GCCGCTAAGAGCACAGCAA	19	TCCCCACTCTGAAAATGAGGA	21
Occludin	TTGAAAGTCCACCTCCTTACAGA	23	CCGGATAAAAAGAGTACGCTGG	22
N-cadherin	AGCGCAGTCTTACCGAAGG	19	TCGCTGCTTTCATACTGAACTTT	23
beta-Catenin	CCCAGTCCTTCACGCAAGAG	20	CATCTAGCGTCTCAGGGAACA	21
Nectin2	GCATCATTGGAGGTATTATCGCT	23	GAGGGAGGTCCTTCCAGTTC	20
beta-Actin	GGCTGTATTCCCCTCCATCG	20	CCAGTTGGTAACAATGCCATGT	22

### Immunoblot analysis

We determined total protein concentration using the BCA method from 20 mg of frozen testicular tissue extracted by RIPA lysate as previously described. Then 60 mg of protein samples were separated by 7.5% SDS-PAGE( Millipore, America) and electrophoretically transferred to PVDF membranes (Millipore, Billerica, MA, USA). The membranes were blocked by Tris-buffered saline supplemented with 0.1% Tween 20 and 5% non-fat dry milk (TBST-milk) for 1 h at room temperature. Following blocking, the membranes were incubated overnight with specific primary antibodies (ZO-1, occludin, N-cadherin, beta-catenin, nectin2, JAM), washed, and then conjugated with secondary antibodies peroxidase-conjugated. The antibodies are shown in Table 2. Our analysis was performed using ImageJ Lab software, and protein levels were normalized to beta-Actin levels.

**Table 2:** Antibodies used in immunoblot analysis.

Antibody	Species	Corporation	Catalog number
ZO-1	Rabbit	ThermoFisher	40-2200
Occludin	Rabbit	ThermoFisher	71-1500
N-Cadherin	Rabbit	ProteinTech Group, Inc	13769-1-AP
Beta-Catenin	Rabbit	ProteinTech Group, Inc	51067-2-AP
Nectin2	Rabbit	Abcam	Ab135246
JAM	Rabbit	ThermoFisher	36-1700
Beta-actin	Mouse	Proteintech	60008-1

### Immunofluorescence

Frozen sections (10 μm) of testis were fixed in Slides with 95% ethanol for 10 s. After washed with PBS, sections were blocked with 5% donkey serum albumin. At 4 °C overnight, sections of tissue were coated with rabbit anti-ZO-1 (1: 25, ThermoFisher 40-2200), followed by incubation with FITC‐conjugated secondary antibodies and DAPI staining for 1 h. Finally, ZO-1 staining of testicular sections was observed by confocal microscopy (Leica, Germany).

### Statistical analysis

All data were presented as the mean ± SEM. One-way ANOVA was used to analyze the difference between the three groups. The data were considered significant when the P value was < 0.05 (*, ^#^) or < 0.01 (**, ^##^). GraphPad Prism 8 was used for statistical analysis.

### Ethical approval

The animal study was reviewed and approved by the Animal Ethics Committee of Shandong Provincial Hospital. The study was carried out in compliance with the ARRIVE guidelines.

## Results

### The reduction of body weight, improvement of abnormalities in glucolipid metabolism and decline in fertility potential through diet control by switching from HFD to ND

**Figure 1:**
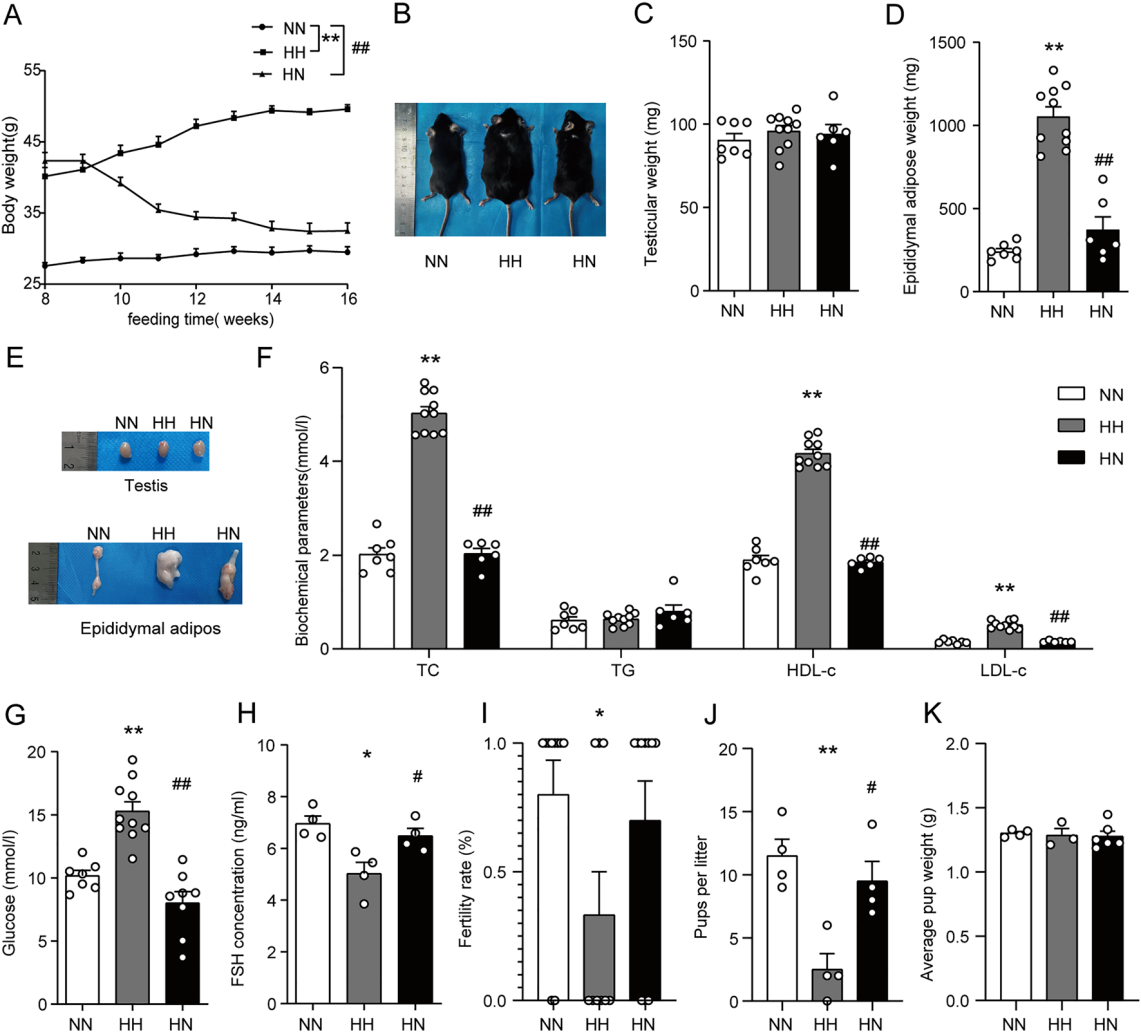
The effects of diet control by switching from HFD to ND on serum lipids, glucose, FSH and fercility potential in HFD-induced obese male mice. (**A**) Comparison of time-dependent changes in body weight among the NN, HH, and HN groups during the 16 weeks of feeding. (**B**) Representative pictures of mice in each group in the 16th week. (**C**) Testicular weight and (**D**) epididymal adipose weight in NN, HH, and HN groups in the 16th week. (**E**) Representative pictures of testis and epididymis in each group in the 16th week. The levels of serum lipid parameters (**F**), serum glucose (**G**), and FSH (**H**) among the NN, HH, and HN groups in the 16th week. Female fertility rate (**I**), pups per litter (**J**), and average pup weight (**K**) were observed at the 16th week of feeding. *NN* normal diet group, *HH* continuous high-fat diet group, *HN* switch from a high-fat diet to a normal diet group. n = 6–10 for each group. *P < 0 05 and **P < 0 01 vs. the NN group; ^#^P < 0 05 and ^##^P < 0 01 vs. the HH group.

To establish whether diet control by switching from HFD to ND could effectively improve male reproductive dysfunction in light of obesity, we found a 16-week HFD-fed obese mouse model and a weight-loss mouse model. Figure 1A,B demonstrate that mice in the HN group lost significant body weight compared to the HH group, considering normal levels reached in the 16th week (P < 0.01). By the 16th week, no difference was observed in testicular weight between the three groups of mice (Fig. 1C). However, the epididymal fat weight was significantly lower in mice switching from HFD to ND (Fig. 1D,E, P < 0.01). These results indicate that diet control by changing from HFD to ND can lower body weight and improve epididymal fat accumulation in obese mice. In addition, Fig. 1F,G illustrate that TC, HDL-c, LDL-c, and Glu were remarkably decreased to normal levels in the HN group (P < 0.01), as opposed to the HH group, suggesting that diet control by switching from HFD to ND could benefit the abnormal glucolipid metabolism caused by HFD.

However, the results highlight the elevation of serum FSH levels in the HN group compared with mice in the HH group (Fig. 1H, P < 0.05). Moreover, in experiments linked with fertility potential testing, the fertility rate of female mice and the number of pups per litter in the HH group were significantly reduced (Fig. 1I, J, P < 0.05). Although there was no significant difference in mean pup weight (Fig. 1K) in the three groups, the number of pups per litter in the HN group was significantly higher compared to the HH group (Fig. 1J, P < 0.05) and the fertility rate of female mice had a tendency to be increased in the HN group (Fig. 1I). According to these results, diet control by switching from HFD to ND can improve obesity-induced abnormalities in FSH level and fertility potential.

### Diet control by switching from HFD to ND can improve testicular lipid deposition and morphological abnormalities caused by HFD

**Figure 2:**
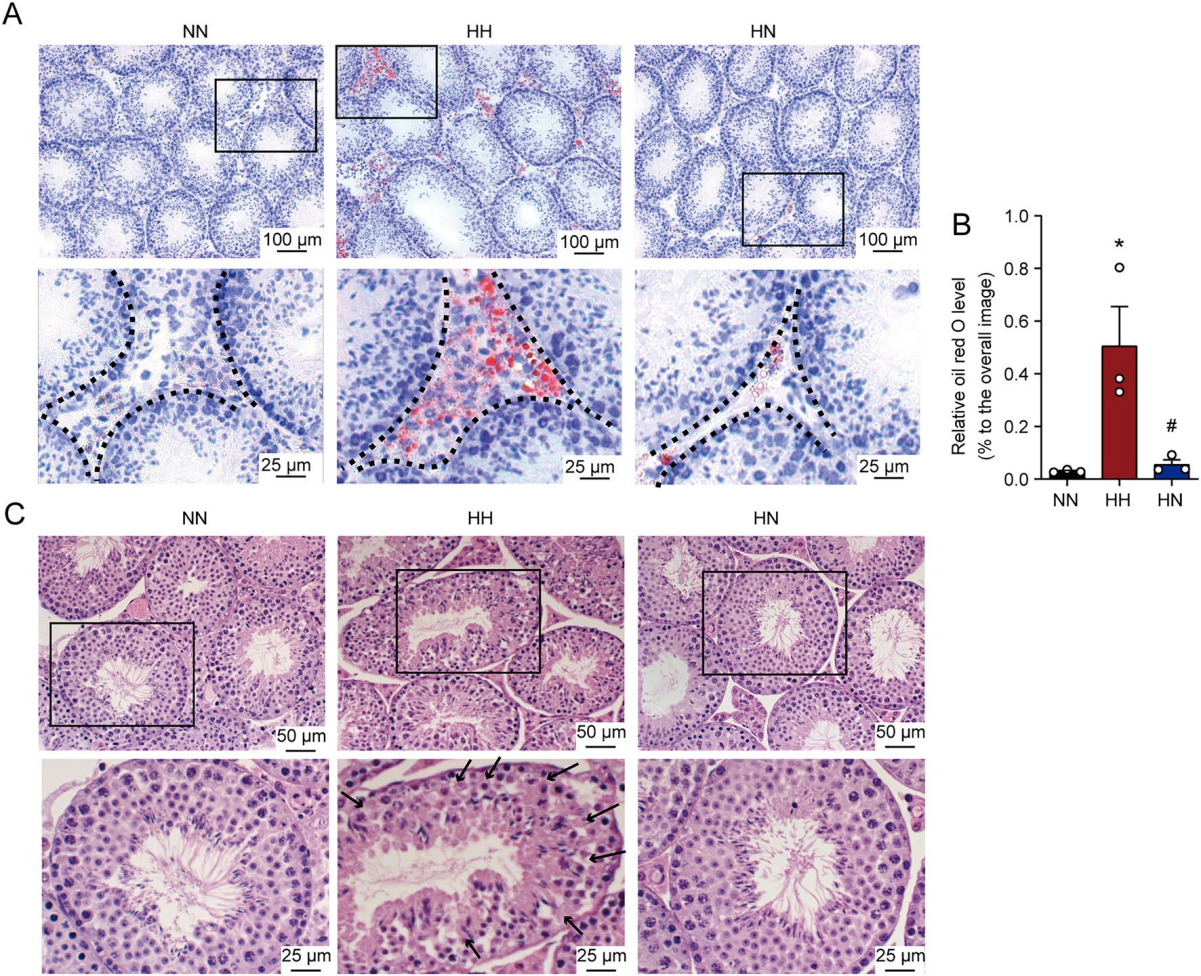
Diet control by switching from HFD to ND alleviates testicular lipid deposition and morphological abnormalities caused by HFD. (**A**) Representative pictures of oil red staining in three groups of mice. The testicular tissues were stained with oil red by the 16th week of feeding. The images in the second row are enlargements of the boxed images in the top rank. (**B**) Lipid abundance in the testis was analyzed. n = 3 for each group. Results are presented as mean ± SEM. *P < 0 05 vs. the NN group; ^#^P < 0 05 vs. the HH group. (**C**) Representative images showing H&E stained testicular sections at the 16th week of feeding among the NN, HH, and HN groups. The images in the second row are enlargements of the boxed images in the top rank and the black arrow represents the morphological change of testicular epithelium.

Oil red O staining showed that HFD greatly enhanced lipid accumulation in the testicular interstitium and seminiferous tubules in the HH group, which was considerably reduced after switching from HFD to ND (Fig. 2A,B, P < 0.05). Notably, the histomorphological changes in the testis were observed with H&E staining. As seen in Fig. 2C, compared with the mice in the NN group, the germinal epithelial organisation of the HH group was severely disorganised, the epithelium was thinned, and the cell adhesion between SCs and germinal cells was disrupted and only loosely arranged. On the contrary, switching to the ND allowed for improvement of the morphology of the germinal epithelium, and the germinal cells were organised in an orderly manner, resembling the morphological structure of the mice in the NN group. Based on the initial results, the HFD disrupts the general morphology of the testis and induces testicular lipid deposition. Therefore, switching to ND can improve testicular lipid deposition and morphological abnormalities of the spermatogenic epithelium.

### Diet control by switching from HFD to ND ameliorates BTB integrity

**Figure 3:**
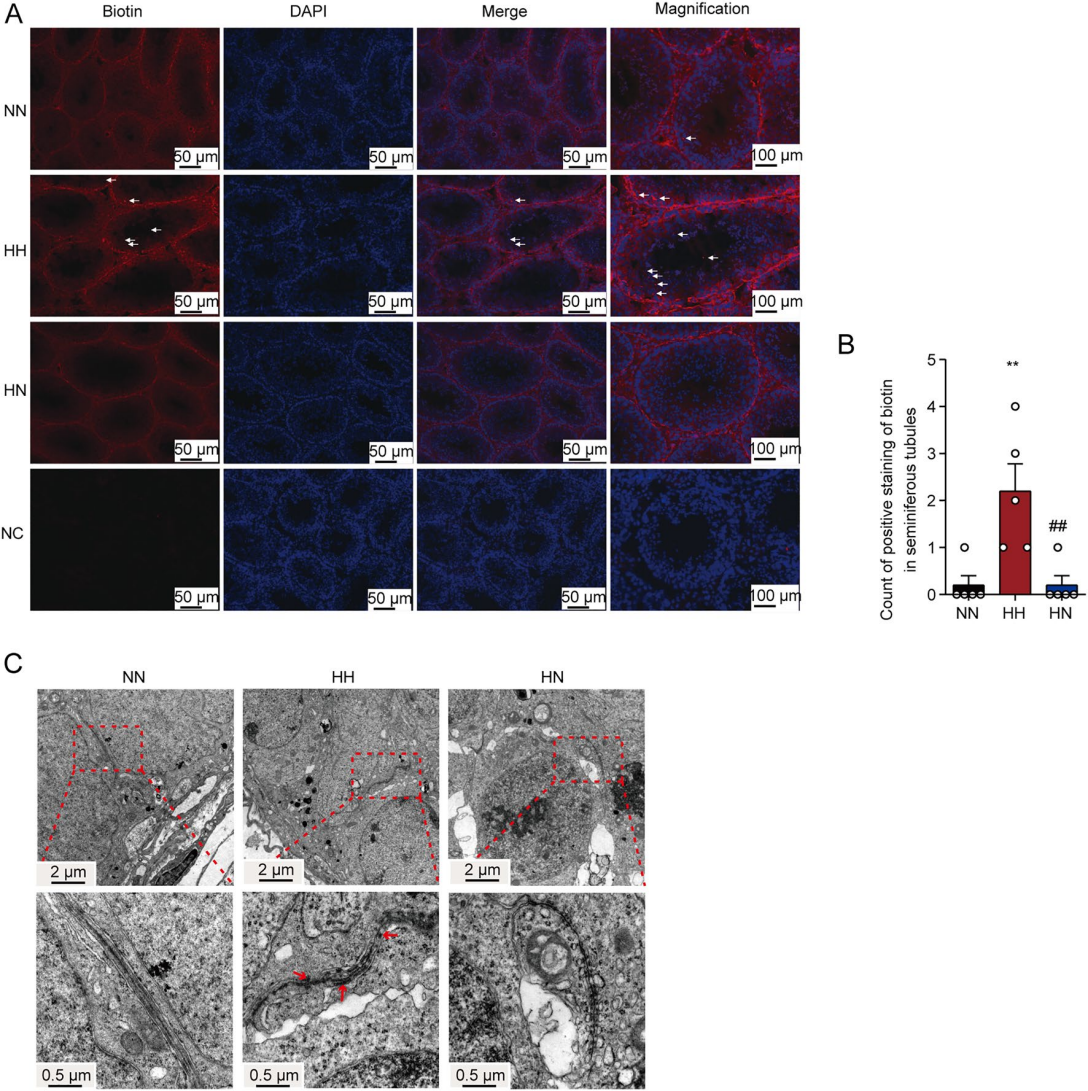
Diet control by switching from HFD to ND ameliorates BTB integrity induced by HFD. (**A**) Representative images showing the Biotin tracer in three groups of mice at the 16th week of feeding. Red fluorescence represents EZ-Link Sulfo-NHS-LC-Biotin and blue fluorescence indicates cell nucleus. NC stands for the negative control group. A large amount of biotin was seen in the lumen of the spermatogenic tubules of the mice in the HH group, as shown by the white arrow. In contrast, the red fluorescence in the NN and NH mice was only in the interstitium and the basal compartment. (**B**) Count of positive staining of biotin in seminiferous tubules between three groups (n = 5 for each group). Results are presented as mean ± SEM. **P < 0 01 vs. the NN group; ^##^P < 0 01vs. the HH group. (**C**) Representative images showing transmission electron micrographs of the germinal Epithelium in each group of mice fed for 16 weeks. The picture at the bottom is an enlargement of each of the above slices. The red arrow indicates the ruptured BTB. The corresponding scales are shown in the lower left corner.

To verify the improvement of BTB integrity by switching to ND, we analysed BTB integrity utilising biotin tracers. As seen in Fig. 3A,B, biotin crossed the BTB and accumulated significantly into the lumens of the majority of seminiferous tubules in the HH group, whereas, in the HN group, biotin was situated solely in the interstitial portion of the testis. The above results illustrate that BTB integrity is disrupted in the testis of mice fed HFD and that switching to ND can ameliorate the integrity disruption.

A testicular TEM analysis was performed to verify further the effect of switching to ND on testicular BTB structure at the ultra-microstructural level. As in Fig. 3C, the testicular Sertoli cells of NN mice were properly arranged with an integrated endoplasmic reticulum (ER) structure and had a typical BTB structure. Nevertheless, in the testis of the HH group, the cell junctions adjoining SCs appeared discontinuous. In addition, they dehisced in the seminiferous tubules, indicating that the integrity of the BTB was impaired. Meanwhile, ER dilation and increased proteins were present on both sides of BTB in the HH group, highlighting abnormal expression of BTB-related proteins and ER stress in mice fed HFD. In contrast, BTB integrity was partially ameliorated in mice switching to ND.

### Diet control by switching from HFD to ND alleviates HFD-induced elevated levels of oxidative stress in the testis

**Figure 4:**
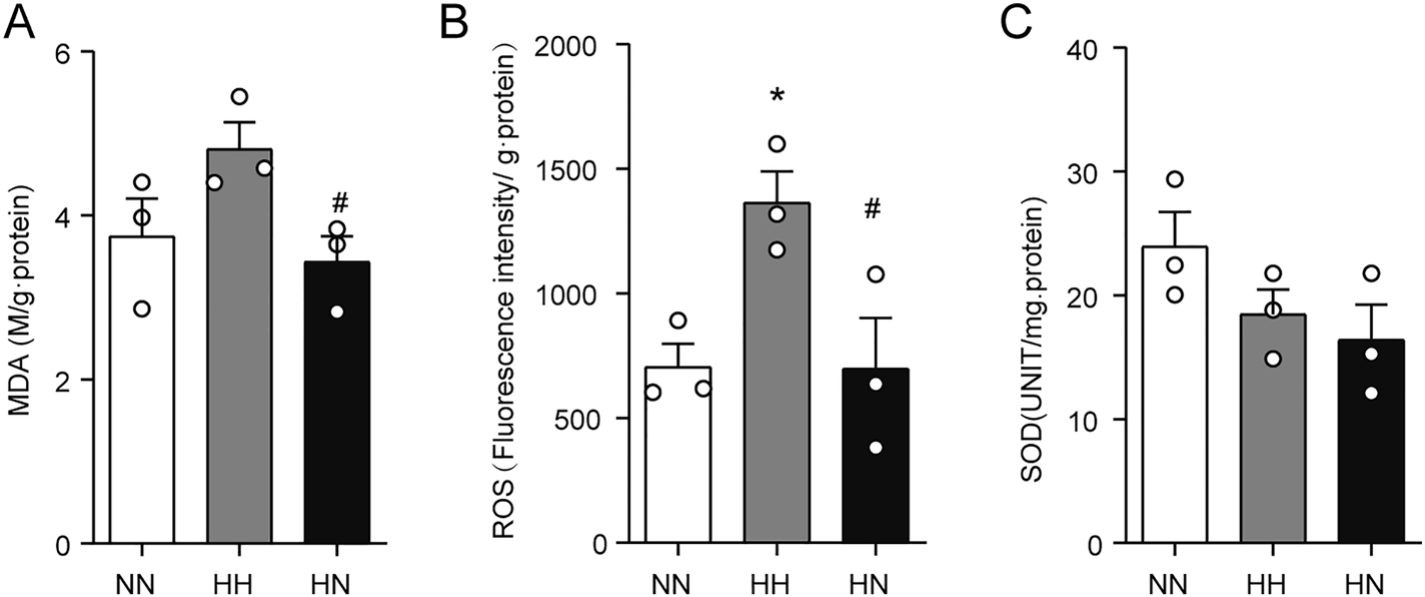
Diet control by switching from HFD to ND reduces the oxidative stress level of the testis induced by HFD. (**A**–**C**) Testicular MDA content, ROS content, and SOD content were assessed at the 16th week of feeding (n = 3 for each group). *P < 0 05 vs. the NN group; ^#^P < 0 05 vs. the HH group.

A range of oxidative stress indicators was examined to assess the effect of diet control by switching from HFD to ND on testicular oxidative stress levels. As seen in Fig. 4, even though no increase in SOD levels was observed in the HH group compared with the NN group (Fig. 4C), ROS levels were remarkably increased in the HH group (Fig. 4B, P < 0.05). On the other hand, the testicular ROS and MDA levels were lowered to normal levels in the mice after switching to ND (Fig. 4A,B, P < 0.05).

### Diet control by switching from HFD to ND ameliorates the abnormal expression of BTB-related proteins induced by the high-fat diet

**Figure 5:**
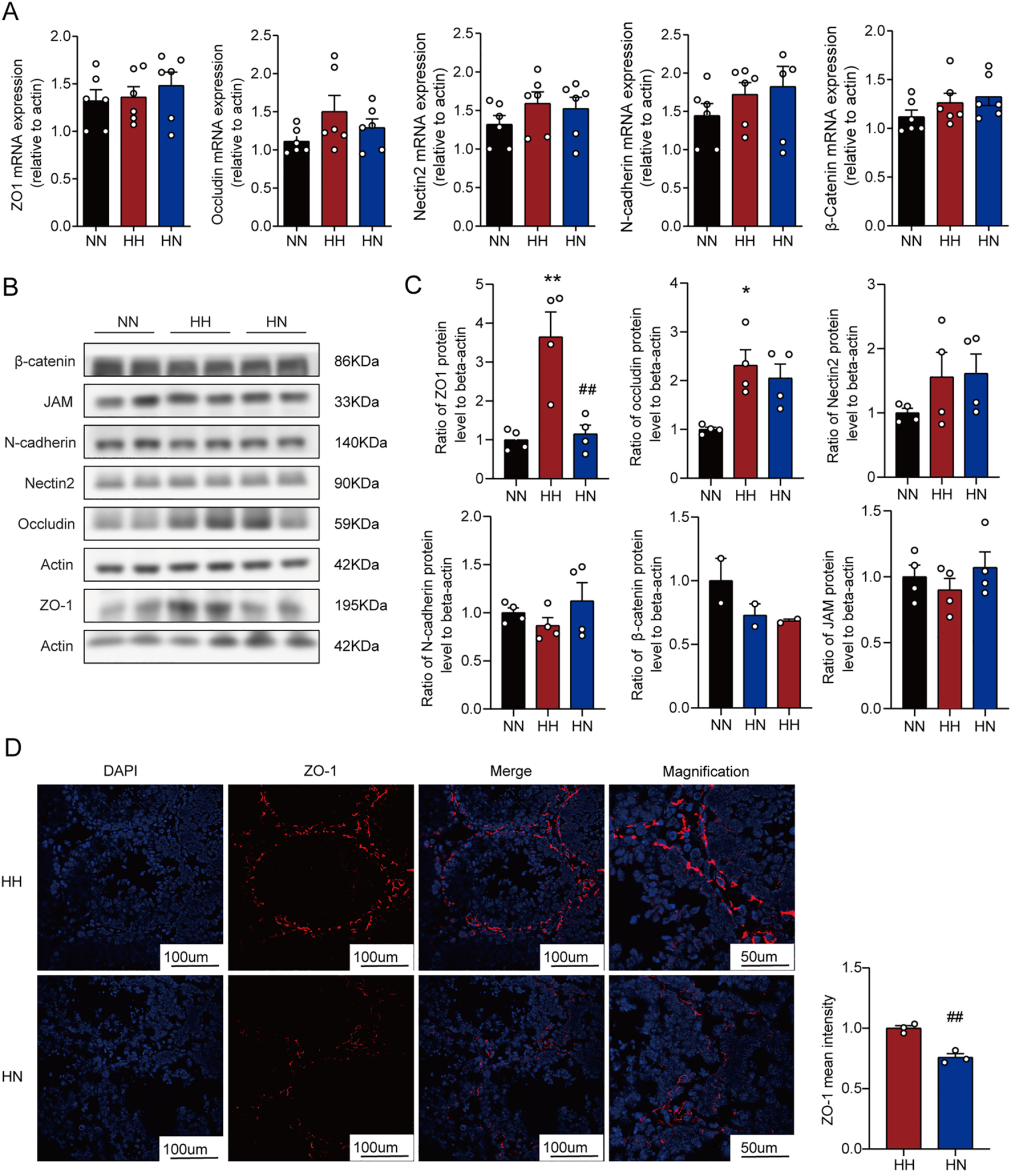
Diet control by switching from HFD to ND ameliorates the abnormal expression of BTB-related proteins induced by HFD. (**A**) Relative mRNA levels of ZO-1, occludin, N-cadherin, β-catenin, and nectin2 were shown by quantification with real-time PCR analysis and normalization to β‐actin levels in the testis. (**B**) Representative bands of Western blotting analysis performed in the testis of ZO-1, occludin, N-cadherin, β-catenin, JAM, and nectin2. The original gels are presented in Supplementary Fig. 2. (**C**) Relative protein density between ZO-1, occludin, N-cadherin, β-catenin, JAM, and nectin2 in three groups. Gene expression was normalized to β-actin. Data were expressed as mean ± SEM. (**D**) Representative images of immunofluorescence staining performed in the testis for ZO-1. Red fluorescence represents ZO-1 and blue fluorescence indicates cell nucleus. Scale bars 50 μm for the magnified images, and scale bars 100 μm for the other images. *P < 0 05 and **P < 0 01 vs. the NN group; ^#^P < 0 05 and ^##^P < 0 01vs. the HH group.

To further assert the mechanism of BTB integrity disruption caused by HFD and the protective effect of switching to ND, the changes in BTB-related molecules at the mRNA and protein levels were considered. The mRNA levels of BTB-related molecules were not significantly altered in the three mice groups (Fig. 5A). However, ZO-1 and occludin proteins were increased drastically in the HH group, and ZO-1 expression was reduced when ND was switched on (Fig. 5B,C). The above results suggest that a high-fat diet induces abnormal BTB protein expression, which could be ameliorated by switching to ND.

Due to the abnormal expression of BTB proteins, the localisation of junction proteins on BTB may affect its function. Therefore, we observed ZO-1 immunofluorescence on BTB to confirm the localization of junction proteins. As seen in Fig. 5D, the localisation of ZO-1 did not differ in the testis of the three groups. Still, consistent with the immunoblotting results, ZO-1 protein accumulated significantly on the BTB in the HH group, and the abnormal accumulation of ZO-1 was diminished after switching to ND.

## Discussion

Obesity and being overweight are associated with low fertility^4,36,37^. Additionally, a convincing association exists between dietary patterns and male fertility, with a low-fat diet benefitting erectile dysfunction and increasing testosterone levels in obese men^15,16^. Calorie and fat restriction is an effective and popular way to reduce obesity^38–41^. Nonetheless, it is uncertain whether controlling fat intake by merely switching from HFD to ND can improve fertility in obese males. The objective of this study was to examine the effects of switching to ND on obese male fertility, as well as the mechanisms involved. According to the results, the high-fat diet mice suffered from reduced fertility potential and had abnormal glucolipid metabolism, impaired BTB integrity, and elevated levels of oxidative stress. Switching to a normal diet improved fertility potential and normalised the glycolipid metabolism of the mice, while oxidative stress levels were lowered and BTB integrity was ameliorated.

High dietary fat content is broadly considered a significant factor in obesity^42^. Furthermore, dietary modification is the cornerstone of weight control, and numerous human studies have reaffirmed that casual consumption of diets with low-fat content can reduce weight in obese individuals^43,44^. This research utilised high-fat and normal diets to feed mice. Both feeds have the same formulation ingredients and total calorie (kcal) content. Only individual formulation ingredients, such as maize starch, maltodextrin, sucrose, and lard, differ regarding calorie contents. The high-fat diets are characterised by fat providing 60% of total calories and protein at normal levels (20% of total calories) to resemble the unreasonably high-fat diet of humans. Simultaneously, normal diets provide only 10% of total calories from fat, 20% from protein, and 60% from carbohydrates.

Sustained HFD-fed mice suffered from weight gain and abnormal glucolipid metabolism, serving as proof of the effective construction of the HFD-induced obesity mouse model. In addition, female mice mated with consistently HFD-fed males had lower conception rates than those mated with the ND group. and the number of pups per litter was significantly lower than those found in ND mice. Furthermore, obese mice switching from HFD to ND lost significant weight from week 9 and returned to normal levels at week 16. The fertility potential of mice switching to ND was greatly ameliorated, as proven by a distinct increase in the number of pups per litter, restored to normal amounts. And there was a trend toward increased fertility in females mated to males returning to a normal diet. These results confirmed the claim that retrieving the high-fat content of the diet to a normal level improves male obesity status and reproductive potential.

A high-fat diet can induce free fatty acid overload and deposition in ectopic tissue, causing structural damage and functional abnormalities^45–47^. To assess the lipotoxicity in the testis, Oil red O and H&E staining were carried out to observe lipid deposition and morphologic changes in the testis of mice. Following dyslipidemia, epididymal fat was remarkably increased in mice fed with an HFD. Testicular lipids are deposited ectopically at the bases of the germinal tubules through the penetration of the basement membrane in the interstitium, which also accumulates a large number of lipids. There was a lack of organisation in the germinal epithelium, and there was a loose arrangement of cell adhesions between SCs and germinal cells. According to these results, the testis and germinal tubules of mice on HFD are in a lipotoxic microenvironment, and the testicular structure was destroyed.

In contrast, after switching from HFD to ND, the mice reduced epididymal fat to normal, followed by significantly improved dyslipidemia. Additionally, lipid deposition in the testicular interstitium was lowered considerably. Testicular spermatogenic epithelial morphology was enhanced, with cell adhesion between SCs and spermatogenic cells returning to normal. These results indicate that returning the high-fat diet to a normal diet significantly benefited the testicular lipotoxic microenvironment and testicular morphology. These can contribute to improving male reproductive potential.

Hormones of the hypothalamus–pituitary–gonadal (HPG) axis help control reproduction, and SCs in the basal compartment of seminiferous tubules play an essential role in testicular development and spermatogenesis, essential for maintaining male fertility^27^. FSH acts on SCs and influences their proliferation, maturity, and function^24–26^. In adult mice, FSH drives SCs to produce regulatory molecules and nutrients necessary for spermatogenesis^27,48^. Furthermore, The FSH regulates structural genes that are critical to organising cell–cell junctions and metabolizing nutrition delivered by SCs to germ cells^27,28^. Our research highlights that FSH levels were greatly reduced in mice on HFD.

Nevertheless, FSH levels were elevated in mice switching to ND. Alterations of FSH propose changes in the HPG axis, possibly affecting fertility in male mice through SCs. Thus, it is recommended that the improvement of fertility potential, testicular spermatogenic epithelial morphology, and adhesion between SCs is attributed, on the one hand, to the alleviation of the lipotoxic state with lowered testicular lipid deposition and, on the other hand, to the restoration of FSH circulating levels, improving the structure and function of SCs.

BTB primarily comprises vascular endothelial cells, germ cells, SCs, and stromal membranes, among which the role of SCs proves of utmost importance^49^. Various junctional structures formed between the SCs, like a tight junction, adherens junction, and gap junction, are the foundation for the maintenance of the structural integrity of the BTB^8,23,50,51^. Deficiencies in BTB integrity harm the reproductive system and cause reproductive dysfunction^20,22^. The previous study confirmed that HFD disrupts BTB integrity, thus impairing male reproductive function^35^. Yet, it remains unclear whether diet control by merely switching from HFD to ND can alleviate BTB damage in obese males to restore their fertility.

As such, the integrity of BTB by transmission electron microscopy and immunofluorescence with biotin tracers was first assessed. The results of electron microscopy indicate that the structure of BTB in HFD mice became blurred, cellular connections adjacent to SCs in the germinal tubules were discontinuous, the ER expanded, and the BTB protein got higher, leading us to believe that mice fed HFD may have ER stress and abnormal expression of BTB-related proteins. Furthermore, biotin accumulated in the glandular compartment of the germinal tubules across the BTB. On the other hand, the mice switching to ND recovered a continuous and compact BTB structure. Furthermore, no biotin accumulation was noted in the glandular lumen of the germinal tubules of mice switching to ND, indicating the improvement of BTB integrity.

Human, as well as mouse experiments, have shown elevated levels of oxidative stress in obese patients^32^. Excessive intracellular mitochondrial ROS secretion is linked with testicular damage and male infertility^52,53^, and ROS-mediated sperm damage is an important cause of infertility in 30–80% of men^33,34^. Previous studies have demonstrated that the activated ROS signaling pathway can add to HFD-induced BTB damage^35^. Other studies have reaffirmed that vitamins C and E reduce BTB damage by alleviating oxidative stress and benefitting male fertility^31,54^. Accordingly, to assess if oxidative stress was involved in BTB damage, we examined the levels of testicular oxidative stress in mice.

Prior studies indicated that protein restriction in dietary control, specifically methionine restriction, is determinant of oxidative stress rather than calorie and fat restriction^55–58^. Nevertheless, our results confirm that ROS levels are significantly higher in HFD-fed mice and that weight loss by switching from HFD to ND to restrict fat intake can lower ROS levels back to normal. In MDA tests, oxygen radicals are detected as a result of attacks on active cells, which reflect the body's level of free radical metabolism and the severity of the attacks. Higher MDA triggers oxidative stress, contributing to cellular damage and even proving to be a cause of death^59,60^. A significant decrease in testicular MAD levels in mice switching to ND was discovered. Superoxide dismutase (SOD) is an antioxidant enzyme responsible for scavenging superoxide anion radicals within a living organism. Our study confirms that SOD levels have a tendency to decrease in mice continuously fed a high-fat diet, implying that there may be insufficient scavenging of ROS. However, SOD levels did not improve in mice switched to a normal diet. A variety of antioxidant enzymes including superoxide dismutase (SOD), catalase (CAT), glutathione peroxidase (GSH-PX), and glutathione reductase (GR) exist in the vivo which constitute the first line of antioxidant defense. The elimination of reactive oxygen species (ROS) is attributed to the collective impact of various antioxidant enzymes. Consequently, we posit that the restoration of ROS levels in mice returned to a normal diet may be attributed to the intricate regulation of multiple antioxidant enzymes. This highlights that weight loss by changing from HFD to ND can significantly reduce testicular oxidative stress levels in obese males, possibly contributing to the improvement of BTB integrity.

The BTB structure at the molecular level primarily comprises various associated and linked proteins. For example, tight Junction includes ZO-1, Occludin, and JAM protein; Basal Ectoplasmic Specialization has N-Cadherin, B-Catenin, and Nectin2^61^. A junction between SCs is where these proteins are located on the membrane of the cell. They are part of forming various connexin complex structures in a dimer or hexamer pattern to keep the normal structure and function of BTB^20^. Yet, under the stimulation of external factors, the expression and localisation of these proteins may undergo abnormal alteration, causing structural damage to BTB^62,63^. Hence, we further examined the changes in BTB-related molecules at the mRNA and protein levels.

Even though there was a lack of change observed in BTB-related molecules at the RNA level in the three groups of mice, TJ-related proteins like ZO-1 and occludin were significantly higher when mice were fed the HFD. Therefore, it is suggested that TJ-related proteins can be targets of lipotoxicity and oxidative stress. This compromises BTB integrity when germinal tubules are in a lipotoxic microenvironment and levels of oxidative stress are elevated. The increase in ZO-1 and occludin proteins is a compensatory response to BTB damage. SCs are synthesising a higher amount of TJ-related proteins to repair the dehiscent BTB, as claimed by the study of Morgan et al.^64^. However, there are also studies confirming that TJ-related proteins are decreased in mice on a high-fat die^11,65^. The different alterations of TJ-related proteins in high-fat diet mice among these contradictory studies may be attributed to the different duration of high-fat diet feeding. Our experiments confirmed a compensatory increase in TJ-associated protein for mice fed a high-fat diet by 16 weeks, but a decline in TJ-related proteins may be observed if high-fat diet feeding is continued for longer durations. In mice switching from HFD to ND, BTB integrity was ameliorated, and the compensatory response of ZO-1 proteins disappeared as testicular lipotoxicity and oxidative stress improved. The current study indicates that diet control by switching from HFD to ND improves the abnormal expression of BTB-related proteins and can be part of ameliorating the structural integrity of BTB.

Normal expression of BTB-related proteins is critical in maintaining BTB integrity and male fertility. Oxidative stress can affect the expression of related proteins. Nevertheless, our experiments did not probe how diet control restored the expression of BTB-associated proteins, keeping the BTB integrity by lowering oxidative stress levels. The relationship between oxidative stress and BTB-related protein and the possible mechanism will be further explored in coming studies. Additionally, the restoration of FSH levels, as well as the alleviation of pituitary lipotoxicity, can contribute to BTB repair. The effect of pituitary lipotoxicity on fertility in obese men and the potential mechanism of the impact on fertility deserve future research.

In sum, diet control by switching from HFD to ND can lead to effective weight loss in obese male mice. Furthermore, A reduction in fat intake can lower oxidative stress in obese male mice's testis, ameliorating the integrity of BTB and enhancing fertility potential. The experiment confirms the rationale for restoring a regular diet to reduce dietary fat content, reasserts the benefit of lowered fat intake in improving fertility potential in obese men, and suggests an effective treatment option for infertility in men suffering from obesity.

### Supplementary Information

Supplementary Information.

## Data Availability

The original contributions presented in the study are included in the article/Supplementary Material. Further inquiries can be directed to the corresponding authors.
