# Supplementary material for: Switching from high-fat diet to normal diet ameliorate BTB integrity and improve fertility potential in obese male mice
Source: Sci Rep. 2023 Aug 29;13:14152. doi: 10.1038/s41598-023-41291-2 (PMC10465505; doi:10.1038/s41598-023-41291-2)
Supplement: Supplementary file 1 — Supplementary Information. [file 41598_2023_41291_MOESM1_ESM.pdf]

Supplementary Material

1. Supplementary Figures and Tables

1.1 Supplementary Figures

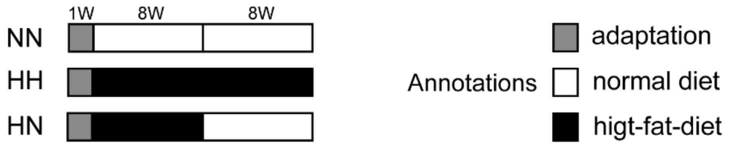

Supplementary Figure 1: The flowchart of the animal experiments.

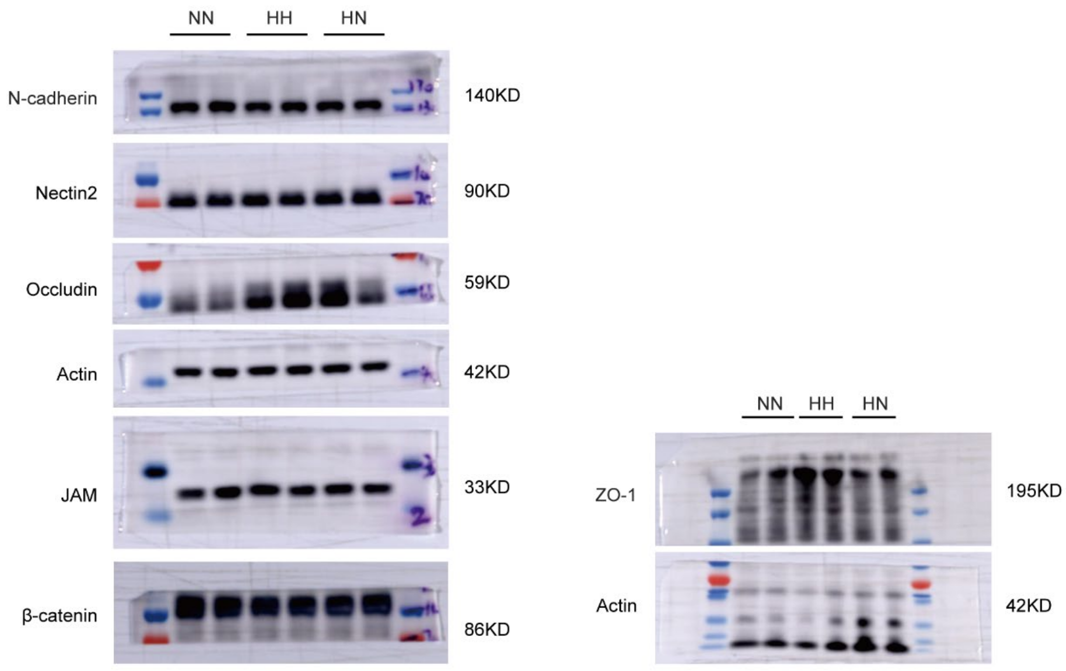

Supplementary Figure 2: The full scan of the entire original blots.

1.2 Supplementary Tables

Supplementary Table 1: Energy supply ratio of experimental diet.

Energy supply material	Normal control diet		High-fat diet	
	gm%	kcal%	gm%	kcal%
Protein	19.2	20	26	20
Carbohydrate	67.3	70	26	20
Fat	4.3	10	35	60
Total		100		100

Reference：https://researchdiets.com/formulas/d12492, https://researchdiets.com /formulas/d12450b
